# Investigating a Telerehabilitation Platform Integrated With a Rehabilitation Robot Using Microsoft HoloLens 2 for Upper-Limb Therapy: Pilot Usability Study

**DOI:** 10.2196/75907

**Published:** 2025-10-22

**Authors:** Md Mahafuzur Rahaman Khan, Md Ishrak Islam Zarif, Aditya Pillai, Inga Wang, Mohammad H Rahman

**Affiliations:** 1 Department of Mechanical Engineering, University of Wisconsin–Milwaukee, 3200 North Cramer Street, Milwaukee, WI, 53211, United States, 1 4148650762; 2 BioRobotics Laboratory, University of Wisconsin–Milwaukee, Milwaukee, WI, United States; 3 Department of Computer Science, Marquette University, Milwaukee, WI, United States; 4 Department of Rehabilitation Sciences & Technology, University of Wisconsin–Milwaukee, Milwaukee, WI, United States

**Keywords:** Azure IoT, Internet of Things, digital twin, HoloLens 2, mixed reality, robot-aided therapy, telerehabilitation, upper-limb dysfunction

## Abstract

**Background:**

Upper-limb impairments following conditions such as stroke and spinal cord injury contribute significantly to long-term disability. Many survivors of stroke face limited access to rehabilitation due to geographical, financial, or scheduling barriers, leaving unmet therapeutic needs.

**Objective:**

This study conducted a preliminary evaluation of the usability of a novel telerehabilitation platform integrating a portable, desktop-mounted robot (DMRbotV3) with a mixed reality HoloLens 2 application to support accessible and adaptive upper-limb neurorehabilitation.

**Methods:**

This was a pilot usability study. Six participants, 3 (50%) stroke survivors (≥3 months after the event) recruited from a hospital stroke registry and 3 (50%) occupational therapists (≥1 year of clinical experience) recruited through convenience sampling in the Greater Milwaukee region, completed a single 2-hour session using the telerehabilitation platform in the BioRobotics laboratory at the University of Wisconsin-Milwaukee. Participants tried out the system, which delivered passive, active, and resistive exercises through DMRbotV3 combined with interactive mixed reality displays, and then completed a customized usability questionnaire. Data collected included (1) robotic parameters from onboard sensors (joint position, velocity, and interaction forces); (2) participant usability feedback from questionnaires to assess usability, satisfaction, and user engagement; and (3) documentation of adverse events and safety concerns. Descriptive statistics (mean scores and ranges) were used to analyze usability ratings and performance parameters.

**Results:**

The system was safe and well tolerated, with no adverse events reported. All participants completed the session, and usability scores averaged ≥4.0 across all items, reflecting high satisfaction and engagement with mixed reality integration. The robotic system demonstrated smooth performance, with controlled joint velocity profiles (–10 m/s to +10 m/s) and adaptive interaction forces up to approximately 25 N.

**Conclusions:**

This preliminary study supports the usability of combining robotics and mixed reality technologies into a telerehabilitation platform for upper-limb neurorehabilitation. Participant feedback also identified opportunities for refinement to enhance adaptability and personalization of therapy.

## Introduction

Upper-limb dysfunction is a frequent consequence of neurological and musculoskeletal conditions such as stroke, spinal cord injury, cerebral palsy, multiple sclerosis, amyotrophic lateral sclerosis, trauma, occupational injuries, and age-related disorders [1]. Among these conditions, stroke accounts for the highest proportion of upper-limb dysfunction cases (approximately 34%), followed by spinal cord injury (27%), multiple sclerosis (19%), and cerebral palsy (8%) [2]. Restoration of upper-limb motor function is essential for performing daily activities, with rehabilitation serving as the cornerstone of functional recovery for patients with upper-limb dysfunction. Conventional rehabilitation typically involves intensive, one-on-one engagement with qualified therapists in clinical settings over extended periods [3]. However, this approach poses significant challenges, including high costs, limited availability of therapy sessions, and logistical barriers such as transportation difficulties, making sustained rehabilitation unattainable for many patients [4,5]. These challenges are further impacted by a shortage of therapists capable of providing personalized, long-term care, particularly in underserved areas [6]. Consequently, many patients often experience premature discharge and insufficient follow-up care, depriving them of the consistent rehabilitation needed for optimal recovery [7].

Robot-aided therapy has demonstrated promise in improving upper-limb rehabilitation outcomes by providing high-intensity, repetitive, and personalized training that supports motor relearning [8-11]. Taveggia et al [12] demonstrated that patients using robotic therapy experienced significantly improved strength, reduced spasticity, and better pain management. Their findings support the use of robotic assistance to alleviate the physical demands on therapists and provide patients with higher-intensity sessions [12]. The integration of machine learning algorithms into robotic systems has further improved the adaptability of these devices. Ai et al [13] reviewed various machine learning applications in robotic upper-limb rehabilitation, finding that algorithms capable of real-time learning can enhance robot-patient interaction, predicting patient needs and adjusting support accordingly. This advance increases the personalization of therapy, aligning it more closely with the fluctuating needs of stroke survivors [13].

The development of technologies for remote rehabilitation has revolutionized the management of poststroke upper-limb impairments, mainly through advancements in telerehabilitation, mixed reality applications, and robotic assistance. Several studies highlight the impact of telerehabilitation on improving access and sustaining engagement among poststroke patients. For example, Housley et al [14] explored telerehabilitation robotics as a promising adjunct to conventional therapies, integrating telehealth and robotic systems to enhance upper-limb function remotely. They found that telerehabilitation significantly increased therapy accessibility and patient adherence, providing an option for continuous care beyond the clinical setting [14]. A notable advancement in telerehabilitation is integrating gamification and remote feedback features. Rodriguez-de-Pablo et al [15] conducted a study on the ArmAssist system, a low-cost robotic system equipped with serious games for upper-limb rehabilitation, which allowed therapists to monitor patient progress remotely. Their findings demonstrated that gamified environments improved patient motivation and engagement and enabled the remote assessment of motor performance, which is crucial for adjusting therapy as patients progress [15]. However, telerehabilitation faces challenges related to communication delays and the limited capacity for real-time, in-depth sensory feedback, which is necessary for more effective motor recovery [16].

Mixed reality technologies, notably Microsoft’s HoloLens 2, have transformed telerehabilitation by creating immersive virtual environments that enhance patient engagement and spatial awareness during physical therapy exercises. MR combines augmented reality and virtual reality (VR), allowing users to interact with holographic projections in real-world settings, which has shown potential in upper-limb rehabilitation by immersing patients in task-oriented training. Palumbo [17] examined HoloLens applications across various health care fields, emphasizing its value in creating interactive, digitally enriched environments conducive to motor rehabilitation. Their study suggested that HoloLens fosters a sense of patient presence and focus, thereby increasing the motivation for continuous practice [17]. Pillai et al [18] implemented a mixed reality–based gamified rehabilitation system on the HoloLens 2 specifically designed to make upper-limb exercises more engaging; their results showed that patients adhered to therapy regimens and improved motor skills [18]. Furthermore, mixed reality technologies in rehabilitation facilitate patient feedback and movement tracking. Vona et al [19] integrated speech-based virtual assistants into mixed reality environments to aid patients in completing rehabilitation exercises, finding that virtual assistants enhanced patient autonomy in home-based settings [19].

Existing commercially available devices such as continuous passive motion devices primarily offer passive therapy, which is limited in adaptability and lacks the active, responsive interactions needed for effective upper-limb rehabilitation in stroke survivors [20]. Robotic rehabilitation devices such as CLEVERarm [21] and Harmony [22] were created to enable home-based rehabilitation; however, these systems are still under development and do not always work well for full-spectrum therapy, limiting their use [23]. Commercially available devices such as ArmeoPower [24], InMotion ARM [25], and ReoGo [26] are mostly used in clinical settings and only support a few motion modes or 2D movements, making them less useful for conducting 3D exercises that target specific joints and are needed for full recovery [27]. Furthermore, many existing systems such as Tyromotion’s AMADEO [28] are designed for specific tasks and lack broad upper-limb training capabilities. Another limitation is the absence of telerehabilitation functions, which restricts these devices to in-clinic use and limits access for patients who require remote support [29].

Recent advancements in home-based telerehabilitation have introduced transformative technologies, including artificial intelligence, VR, and digital twin (DT) frameworks, which offer adaptive and interactive solutions tailored to individual patient needs. VR-based systems integrated with collaborative robotics have significantly enhanced patient engagement and therapeutic outcomes through gamified exercises that promote active participation [30]. Home-based platforms such as the telepresence rehabilitation system have demonstrated the potential for remote therapy by incorporating therapist-in-the-loop designs with VR-enhanced environments and haptic feedback for interactive training [31]. Systems such as GARMI exemplify the seamless integration of robotics with telemedicine by offering dynamic and adaptive rehabilitation modes supported by optimal control and game theory, along with user-friendly interfaces and real-time communication features [32]. Similarly, the MERLIN system leverages serious gaming to boost patient engagement, although ergonomic challenges affect user satisfaction [33]. Other systems, such as the PVSED exoskeleton [34] and Hand Mentor Pro [35], offer advanced haptic feedback and innovative training modules but face limitations related to cost, portability, and accessibility. DT technology further facilitates remote monitoring and real-time customization of therapy, enabling therapists to deliver highly personalized interventions [36,37]. Similarly, the xArm-5 integrates industrial Internet of Things (IoT) and augmented reality to deliver remote therapy programs with real-time visualization and feedback [37]. Despite these advancements, key challenges remain, including the absence of seamless real-time data transfer and visualization and the inability to offer passive, active, and resistive therapies that deliver personalized and adaptive treatment remotely.

The aim of this study was to conduct a preliminary evaluation of the usability of a novel telerehabilitation platform integrating a portable, desktop-mounted robot (DMRbotV3) with a mixed reality HoloLens 2 application to support accessible and adaptive upper-limb neurorehabilitation. Participants interacted with the device (tryout session) and provided feedback through a customized usability questionnaire to assess usability of and satisfaction and user engagement with the system.

## Methods

### Study Sample

To evaluate the usability of the device, two participant groups were recruited: (1) survivors of stroke, representing the end users who would directly engage with the upper-limb rehabilitation system; and (2) occupational therapists, representing the potential operators and clinical supervisors who may adopt the device for rehabilitation in future practice. Survivors of stroke were recruited from the Stroke Survivor Recruitment Database at Froedtert Hospital (Milwaukee, Wisconsin). Eligible participants were required to be aged ≥18 years at the time of enrollment, have had a radiologically confirmed stroke due to ischemia or intracerebral hemorrhage, and have experienced stroke onset more than 3 months before recruitment. Occupational therapists were recruited through convenience sampling in the Greater Milwaukee area. Eligible therapists were required to have at least one year of professional rehabilitation experience.

### System Trial and Feedback Loop

Each participant completed a single 2-hour session in the BioRobotics laboratory at the University of Wisconsin–Milwaukee using the telerehabilitation platform, where they first interacted with the device in a tryout session and then provided structured feedback through a customized questionnaire assessing the usability of and satisfaction and engagement with the system.

**Figure 1.**
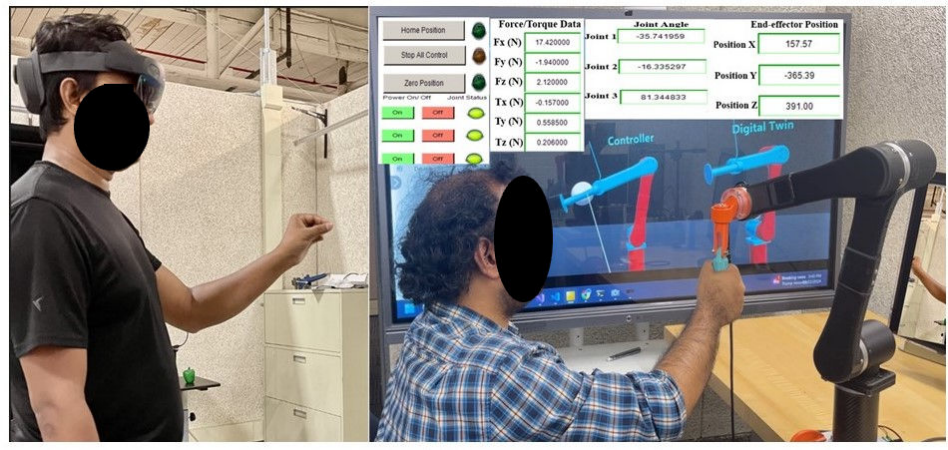
Therapist controlling DMRbotV3 via the HoloLens 2 mixed reality interface, accessing real-time robot data, the participant’s view, and a video call for seamless therapy interaction.

Each session began with an introduction to the telerehabilitation platform, and participants were familiarized with the robotic rehabilitation system and mixed reality interface. Participants were seated in a chair and positioned to hold the DMRbotV3 handle while the therapist controlled the system using a HoloLens 2 mixed reality interface, as shown in Figures1  2, to ensure that the rehabilitation routine was aligned with individual needs and safety requirements. The therapy commenced with passive exercises, where the operator (the person controlling the device) guided the participant’s arm through controlled motions tailored to their range of motion to enhance joint mobility with minimal exertion. Subsequently, participants wore the HoloLens 2 to engage in mixed reality–based active game levels designed to simulate real-world tasks such as reaching for objects and performing repetitive hand movements, as shown in Figure 3. Depending on their abilities, participants performed active tasks with or without robotic assistance. The operator dynamically adjusted resistance levels to match the participant’s progress, adding appropriate challenges to the tasks and enabling strength training through controlled resistance adjustments via the robotic rehabilitation system. Throughout the session, the operator monitored real-time data, including force exertion, robotic parameters, hand positioning, and motion trajectories, to adapt therapy settings for optimal challenge and safety. These metrics were recorded in real time and transmitted via a cloud-based system, allowing for detailed analysis of the participant’s performance and interaction with the system. Real-time feedback was provided via live video communication, guiding participants on posture, movement quality, and task execution.

**Figure 2.**
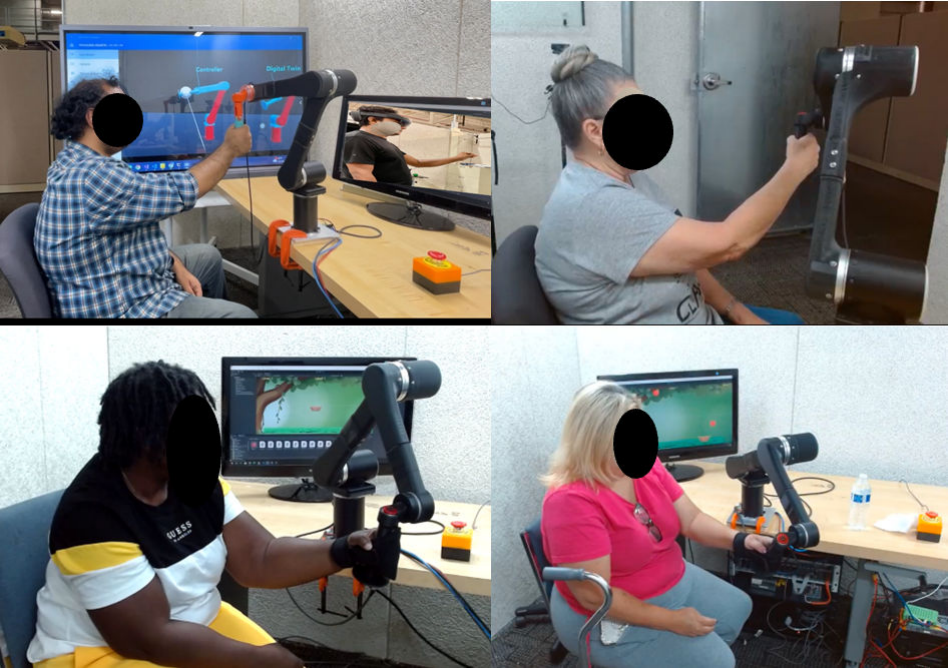
Participants in a remote therapy session within a simulated home environment, communicating with the therapist via video call to provide real-time feedback on the therapy session.

**Figure 3.**
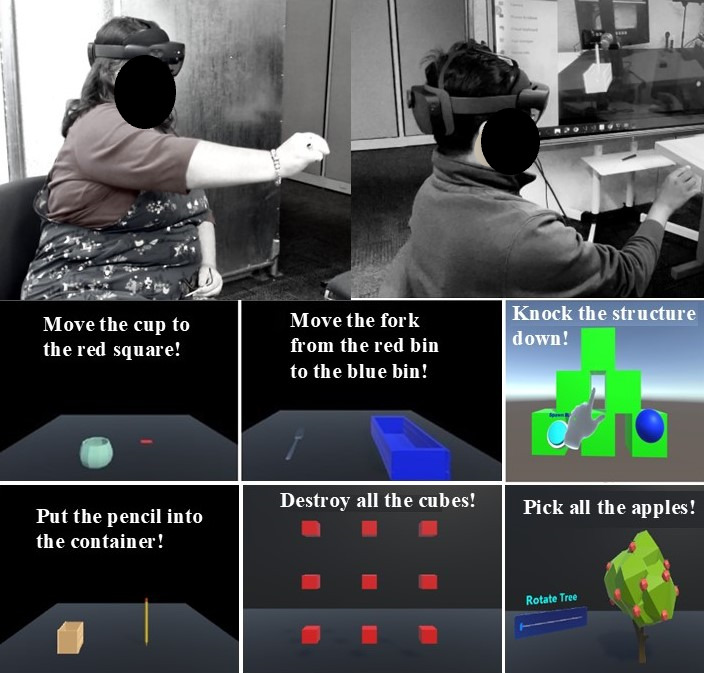
Participants actively engaged in therapy sessions, using the HoloLens 2 to progress through various game levels.

After the tryout session, participants completed a customized 15-item usability questionnaire using a 5-point Likert scale to evaluate the platform’s ease of use, usability, satisfaction, and user engagement. Items assessed multiple domains, including perceived effectiveness and usefulness, comfort and satisfaction with adjustability, and flexibility of use. Additional items focused on ease of use, such as following instructions, performing exercises, personalizing settings, and operating controls, as well as safety perceptions. Items were rated on a 5-point Likert scale (1=“strongly disagree”; 5=“strongly agree”). Established usability questionnaires were not adopted fully because several items were not directly relevant to the unique features and operational demands of an upper-limb robotic rehabilitation device.

In addition, participants provided subjective verbal feedback during sessions and engaged in open discussions with the operator. Integrating these qualitative insights with quantitative measures captured both the system’s technical performance and user experience, yielding actionable feedback to refine the telerehabilitation platform for future applications.

### Overview of the Telerehabilitation Platform

The architecture of the telerehabilitation platform consists of four main components: (1) hardware elements, including the DMRbotV3 and HoloLens 2; (2) software applications for mixed reality interaction and therapy control; (3) cloud-based IoT communication and data storage infrastructure; and (4) the exercise module, including passive, active, and resistive therapy games designed to adapt to user needs and deliver personalized upper-limb rehabilitation.

#### Hardware Elements

The telerehabilitation platform integrates a DMRbotV3 with a mixed reality HoloLens 2 application to deliver personalized therapy in a remote format. The configuration includes a mixed reality interface that enables precise control of the robotic rehabilitation system, real-time monitoring of therapy sessions, guidance of the DMRbotV3, and on-the-fly adjustments to therapy (exercise games) parameters. Specifically, the DMRbotV3 is a portable rehabilitation robot specifically designed for delivering physical therapy exercises. Equipped with a force sensor in its handle, it provides real-time feedback on the patient’s arm stiffness, which helps the therapist assess and adjust exercises remotely. This force feedback enhances therapy customization and supports adaptive rehabilitation by allowing therapists to monitor patient response actively during sessions. The DMRbotV3 administers passive, active, and resistive therapy, whereas video communication tools support continuous feedback and instruction.

The HoloLens 2 [38] serves as a vital component of the telerehabilitation platform, providing an advanced mixed reality interface that enables immersive and hands-free rehabilitation. With its sophisticated sensors for hand tracking, eye tracking, and depth sensing, the device allows patients to interact with holographic guides and video game levels that provide precise instructions for therapeutic exercises. The headset can seamlessly integrate real-time feedback from the platform’s data systems and offer immediate visual insights on metrics such as joint angles and range of motion, helping patients refine their movements for optimal rehabilitation outcomes. By incorporating gamified elements such as timed objectives and progress indicators, HoloLens 2 enhances engagement and motivation, making it an indispensable tool for effective remote rehabilitation.

#### Software Applications

Unity (Unity Technologies) is used to create the mixed reality environment and support 3D model import, enabling immersive user interfaces. The TwinCAT software (Beckhoff Automation) running on a PC interfaces with the DMRbotV3 to execute control commands and stream telemetry data. In addition, a Mixed Reality Toolkit 3 (MRTK3; Microsoft Corp)–based application on the HoloLens 2 manages therapy modes, facilitates user interaction, and provides real-time visualization.

Unity and Visual Studio (Microsoft Corp) [39] are essential components of the telerehabilitation platform, working together effectively to enable the development and deployment of immersive mixed reality applications. Unity, a versatile and powerful game engine, serves as the foundation for designing dynamic and interactive rehabilitation environments, integrating holographic elements with real-world interactions to emulate engaging user experiences. Visual Studio, a robust integrated development environment, enhances this process with advanced coding, debugging, and deployment tools optimized for HoloLens 2 compatibility. The synergy between Unity and Visual Studio ensures a streamlined workflow, allowing developers to build responsive and user-friendly applications that meet the platform’s requirements for precision, adaptability, and patient engagement.

MRTK3 [40] is a critical component of the telerehabilitation platform, providing the foundational framework for developing immersive and interactive applications on HoloLens 2. This open-source toolkit from Microsoft offers a robust set of features, including prebuilt user interface elements, game objects, and scripts that enable robust interaction between patients and their mixed reality environment. MRTK3 simplifies the development process and allows for the creation of engaging, user-friendly interfaces tailored to rehabilitation needs. Its flexibility and customization options make it an indispensable tool for building gamified applications that improve patient engagement, ensure ease of use, and support the dynamic requirements of telerehabilitation.

#### Cloud-Based IoT Infrastructure and Data Storage

**Figure 4.**
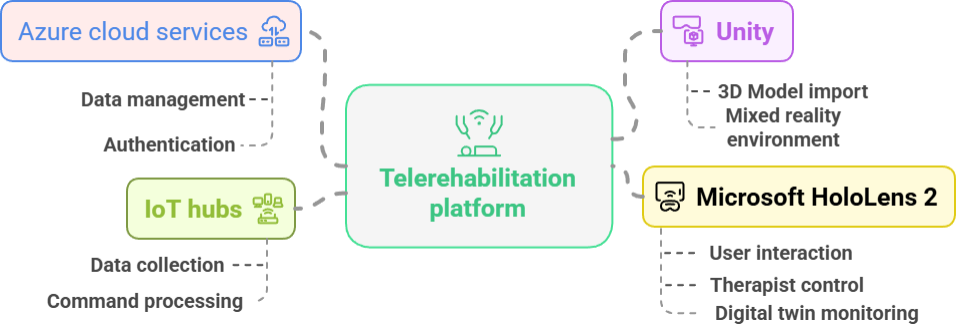
Conceptual architecture of the Azure IoT–enabled telerehabilitation platform with Microsoft HoloLens 2 and Azure services.

The telerehabilitation platform uses a cloud-connected IoT architecture built on Microsoft Azure to enable secure, real-time communication; data storage; and dynamic therapy adaptation. Azure IoT Hub serves as the central gateway for bidirectional messaging between the HoloLens 2 application and the DMRbotV3. When therapy commands are issued through the HoloLens 2 mixed reality interface, they are transmitted via Azure IoT Hub to a TwinCAT PC, which drives the robotic arm’s therapeutic actions. Conversely, telemetry from the DMRbotV3, including joint states, force feedback, and movement data, is streamed back to Azure IoT Hub and securely logged in Azure Storage. This continuous data flow supports both live visualization on the HoloLens 2 interface and the creation of a DT of the DMRbotV3 within Unity, allowing therapists to monitor performance, adjust therapy parameters in real time, and ensure precise execution of exercises. By integrating authentication, data management, and session logging, the Azure cloud infrastructure provides a reliable foundation for adaptive, safe, and personalized upper-limb rehabilitation (Figure 4).

**Figure 5.**
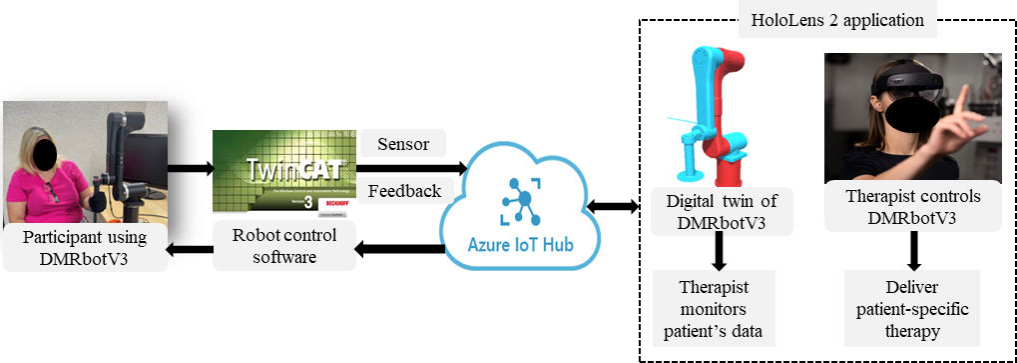
Data flow diagram illustrating the integration of a robotic rehabilitation system with a telerehabilitation platform. IoT: Internet of Things.

A key feature of this system is the DT, which links a 3D model of the DMRbotV3 (developed in Unity) to live telemetry from the Azure IoT Hub. This integration allows for real-time synchronization between the physical robot and its virtual counterpart, enabling therapists to visualize the device’s state, monitor patient performance, and adjust therapy parameters dynamically. By leveraging Azure’s authentication, messaging, and storage services, the telerehabilitation platform cloud framework ensures seamless connectivity between operator and patient endpoints while maintaining high standards of data security, responsiveness, and clinical reliability (Figure 5).

### System Development Summary

In summary, the telerehabilitation platform integrates multiple hardware, software, and cloud components into a seamless rehabilitation system. The DMRbotV3 serves as the robotic hardware delivering passive, active, and resistive exercise modules. The Microsoft HoloLens 2, running an MRTK3-based application, provides the mixed reality interface for therapist control, patient interaction, and visualization. This application is built in Unity, which also supports 3D model import, immersive environments, and integration of the DT of the DMRbotV3. The TwinCAT PC acts as the local controller, executing commands received from the HoloLens 2 via the Azure IoT Hub and streaming telemetry (forces and joint states) back through the same channel. Azure Cloud Services, including Azure Storage, manages authentication, secure data transfer, and session logging. The DT links Unity with live IoT telemetry, enabling real-time monitoring and adaptive adjustments. Finally, the exercise modules, covering passive, active, and resistive therapy, were developed within Unity and deployed through the HoloLens 2 application, ensuring synchronized execution by the DMRbotV3.

### Therapy Modalities

#### Passive Therapy

Passive therapy is crucial for managing movement limitations and motor dysfunctions in survivors of stroke using therapist- or device-assisted movements to maintain joint flexibility and muscle elasticity while preventing stiffness and contractures. Techniques such as passive range-of-motion exercises help preserve mobility and reduce muscle atrophy, supporting long-term recovery potential [41]. The telerehabilitation platform leverages a HoloLens 2 control to implement passive therapy effectively for patients with stroke. Through HoloLens 2 control, operators (or therapists controlling the device) can manually guide the DMRbotV3 to perform specific passive range-of-motion stretching exercises, precisely adjusting the speed, direction, and range of motion. This control enables therapists to customize movements to the patient’s needs, allowing for slow, controlled exercises that accommodate limited range of motion in the early stages of recovery. For remote sessions, the HoloLens 2 mixed reality interface offers a hands-free, interactive alternative where therapists can adjust the DMRbotV3’s trajectory using holographic controls. This approach ensures that patients experience safe, consistent, and responsive movement guidance tailored to their rehabilitative needs.

#### Active Therapy

Active therapy is a critical component of poststroke recovery, enhancing motor control, functional independence, and psychological well-being through patient-driven movements [42]. The telerehabilitation platform incorporates MRTK3 game levels to facilitate active therapy, offering an engaging and interactive experience that motivates patients to participate in their rehabilitation either independently or with the assistance of DMRbotV3. This patient-centric mode includes 2 types of levels. The *eye–activities of daily living* levels involve practical activities such as moving a pencil, fork, and cup to designated areas in a virtual environment and simulating real-life actions to help patients practice essential movements. The *challenge* levels introduce playful elements such as picking apples from a tree, shooting targets, and knocking down structures with virtual objects in the HoloLens 2 environment, making therapy more enjoyable. These MRTK3 game levels are carefully tailored to the patient’s abilities and designed to replicate real-world tasks such as reaching, grasping, and manipulating objects, fostering functional recovery through gamified interventions [18]. In this mode, patients rely on their own abilities to complete exercises, with the game levels dynamically changing based on their level completion. The mixed reality interface provides visual feedback, reinforcing proper movement patterns and enabling patients to monitor their progress. The game levels provide a structured progression that allows patients to start with basic tasks and advance to more complex movements as their abilities improve.

#### Resistive Therapy

Resistive therapy, a strength training approach that incorporates resistance against muscle contractions, is a critical component of poststroke rehabilitation [43]. In the telerehabilitation platform, resistive therapy is implemented through MRTK3 game levels, allowing patients to engage in familiar, interactive tasks while the DMRbotV3 applies moderate resistance to motion. This resistance requires patients to exert more force, actively engaging their muscles as they complete each task. By making the exercises progressively more challenging (changing the resistance level), the DMRbotV3 fosters muscle growth and improves functional strength, preparing patients for more physically demanding activities. The adjustable resistance levels allow for the provision of a tailored strength-building regimen aligned with each patient’s specific recovery goals and capabilities [44].

### Data Analysis

Data from three sources were analyzed: (1) sensor-based performance metrics from the DMRbotV3, including joint position, velocity, and interaction forces; (2) self-reported usability feedback from a customized usability questionnaire; and (3) observation of adverse events or concerns.

Sensor data were summarized using descriptive statistics (eg, means and ranges) to characterize the robotic system’s performance during passive, active, and resistive exercises. Usability questionnaire responses were analyzed using descriptive statistics, with item-level mean scores and ranges calculated to evaluate overall usability and user satisfaction. Safety data were reviewed qualitatively to determine whether any adverse events or safety concerns arose during the study session.

The Mann-Whitney *U* test was applied as an exploratory analysis to compare usability scores between the groups of survivors of stroke and therapists.

### Ethical Considerations

This study was approved by the Institutional Review Board at the University of Wisconsin–Milwaukee (application 22.220). All procedures complied with the ethical principles outlined in the Declaration of Helsinki. Written informed consent was obtained from all participants prior to enrollment following a thorough explanation of the study objectives, procedures, potential risks, and benefits. Participants were assured of confidentiality and their right to withdraw at any time without consequence.

## Results

### Sample Characteristics

This study included 6 participants: 3 (50%) stroke survivors and 3 (50%) occupational therapists. Survivors of stroke ranged in age from 57 to 73 years (all female), had had right- or left-brain stroke, had varying levels of education (elementary school to university), and were from diverse racial backgrounds (Hispanic and African American). Occupational therapists were younger (aged 23‐29 years), represented both genders, and had education at the university or graduate level. Their racial backgrounds included Asian and non-Hispanic White. Table 1 provides the demographic characteristics of the study sample.

**Table 1.** Demographic data of the participants.

ID^a^	Age (y)	Side of the brain affected	Sex	Dominant hand	Educational level	Race
PS1	57	Right	Female	Left	University	Hispanic
PS2	73	Left	Female	Right	Elementary school	Hispanic
PS3	69	Left	Female	Right	High school	African American
OT1	29	—^b^	Male	Right	Graduate school	Asian
OT2	23	—	Female	Right	University	Non-Hispanic White
OT3	24	—	Female	Right	University	Asian

a “PS” refers to patients with stroke, and “OT” refers to occupational therapists.

b Not applicable.

### Robot Parameters

**Figure 6.**
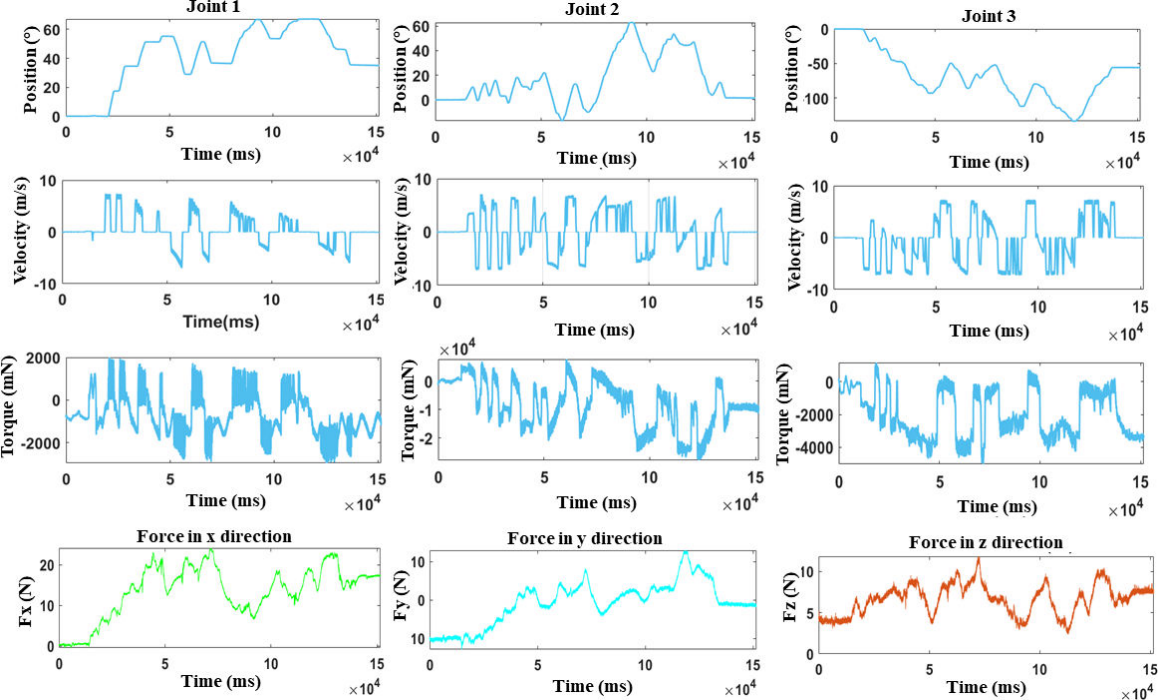
DMRbotV3’s joint position, velocity, torque, and participant force data profiles during HoloLens 2 control for multijoint upper-limb exercises, showcasing smooth robotic movements.

The robot metrics, which include joint position, velocity, and torque, demonstrate the system’s smooth and accurate performance during the multijoint upper-limb workout (Figure 6). Positions for joint 1, joint 2, and joint 3 varied from 0° to 65°, 0° to 60°, and 0 to −120°, indicating a regulated and acceptable range of motion for therapeutic purposes. Velocity profiles across the 3 joints showed comparable oscillatory patterns, with joint 1 to joint 3 functioning in the −10 to +10 m/s range, demonstrating dynamic flexibility. The torque data showed that joint 1 and joint 3 maintained torque levels ranging from −2000 to +2000 mN, but joint 2 had larger torque changes between −4000 and +4000 mN, indicating its function in managing more strenuous lateral and stabilizing activities. These robotic parameters supported the system’s capacity to move precisely, steadily, and smoothly, indicating its appropriateness for therapeutic applications.

The participant force data collected in the x-, y-, and z-axes showed active engagement and physical interaction with the robotic system. Forces applied in the x direction varied from 0 to +25 N, showing that participants exerted substantial effort when moving forward and backward. Forces in the y direction ranged from −10 N to +12 N, representing dynamic lateral motion, whereas forces in the z direction ranged from 0 to +15 N, indicating modest participation in vertical motions such as raising or lowering. The oscillatory patterns in all force directions demonstrated persistent participation, which is essential for motor recovery and strengthening. These results show that the system may provide robot-assisted motions, which are critical for accomplishing rehabilitation goals, including increased strength, coordination, and range of motion.

### Participant Usability Feedback

Overall, mean scores across items ranged from 3.66 to 4.83 (average SD 0.70), indicating high levels of usability, satisfaction, and perceived safety (Table 2). Participants provided consistently high ratings for the device’s effectiveness and usefulness, with mean scores of 4.66 (SD 0.51) for assisting rehabilitation needs (question 1) and 4.83 (SD 0.40) for its overall usefulness (question 2). These findings indicate a strong confidence in the device’s ability to support rehabilitation goals. Comfort during rehabilitation sessions (question 3; mean 4.00, SD 1.26) and satisfaction with the device’s range of motion and adjustability (question 4; mean 4.66, SD 0.51) were also highly rated, reflecting the device’s capacity to accommodate a variety of movement requirements effectively.

**Table 2.** Experience evaluation questions, including the mean, SD, and *P* value for each item response from all participants, with each score ranging from 0 to 5.

Item	Question	Score, mean (SD)	*P* value
1	“How effective do you feel the rehab device is in assisting your rehabilitation needs?”	4.66 (0.51)	.60
2	“How useful do you think this rehab device is?”	4.83 (0.40)	.26
3	“How comfortable did you feel using the rehab device during your rehabilitation sessions?”	4.00 (1.26)	.44
4	“How satisfied are you with the range of motion and adjustability provided by the rehab device during your rehabilitation exercises?”	4.66 (0.51)	.60
5	“How would you rate the durability and reliability of the rehab device for long-term use in your rehabilitation journey?”	4.50 (0.54)	.60
6	“How do you rate the flexibility of using this rehab device?”	4.16 (0.75)	.26
7	“How easy was it to follow the instructions provided while using the rehab robot?”	4.66 (0.51)	>.99
8	“How easy was it to engage in the upper limb exercises facilitated by the rehab robot?”	4.50 (0.54)	.69
9	“How easy was it to personalize the settings and parameters of the rehab robot to suit your needs?”	3.66 (1.21)	.26
10	“How easy was it to operate the controls and interfaces of the rehab robot?”	4.50 (0.54)	.09
11	“How easy was it to adjust the rehab robot’s range of motion or movement patterns?”	4.66 (0.51)	>.99
12	“How easy was it to adjust the resistance levels of the rehab robot according to your abilities?”	4.33 (0.51)	.60
13	“How would you rate the ease of use and user-friendliness of the rehab device?”	4.50 (0.54)	.69
14	“How do you rate the safety of this rehab device?”	4.33 (1.21)	>.99
15	“How comfortable did you feel using the rehab device during your rehabilitation sessions?”	4.23 (0.89)	.55

The durability and reliability of the device for long-term use were favorably perceived, with a mean score of 4.50 (SD 0.54), highlighting participants’ trust in the device’s construction for extended use. The flexibility (question 6; mean 4.16, SD 0.75) and user-friendliness (question 13; mean 4.50, SD 0.54) scores further underscored its ability to cater to diverse user needs. The device’s controls and interfaces were deemed easy to operate (question 10; mean 4.50, SD 0.54), although some participants indicated that personalizing the settings (question 9; mean 3.66, SD 1.21) could require additional support or training. The device’s adjustability for movement patterns (question 11; mean 4.66, SD 0.51) and resistance levels (question 12; mean 4.33, SD 0.51) was positively rated, demonstrating its adaptability to different rehabilitation exercises and user abilities. In addition, participants expressed high satisfaction with the device’s safety (question 14; mean 4.33, SD 1.21) and overall comfort during use (question 15; mean 4.23, SD 0.89), indicating trust and comfort during rehabilitation sessions.

## Discussion

### Principal Findings

This study conducted a preliminary evaluation of the usability of a telerehabilitation platform in upper-limb neurorehabilitation. Overall, the results indicated that the system was safe and well tolerated, with no adverse events reported. All participants completed the session, and usability scores averaged ≥4.0 across all items, reflecting high satisfaction and strong engagement with mixed reality integration. The robot demonstrated smooth joint movements within a defined speed range in both directions and could apply resisting forces up to approximately 25 N (2.5 kg). In practice, this means that, if a patient is weak, the robot provides greater assistance, whereas if the patient is strong, it increases resistance to provide a greater challenge. This adaptability is critical for progressive therapy, ensuring that patients are neither under- nor overchallenged.

This study investigated the integration of the DMRbotV3 telerehabilitation platform with HoloLens 2, focusing on its potential to provide upper-limb therapy to survivors of stroke. The findings highlight the transformational effects of merging mixed reality technology with a robotic rehabilitation system, notably increasing patient involvement and encouraging autonomy during therapy. By allowing for personalized therapies in home-based settings, the system solves essential obstacles such as geographical constraints and restricted mobility, considerably enhancing accessibility. Participants expressed great satisfaction with the system’s operation, as evidenced by an exceptional experience assessment score, and emphasized its potential to aid in long-term rehabilitation. The DMRbotV3 system demonstrated exceptional safety, with no adverse incidents reported. Although this study was conducted under therapist supervision in a simulated home environment and did not test unsupervised self-installation, the good performance observed of the system underscores that prioritizing safety (eg, bounded workspaces, responsive stops, and clear feedback) is central to designing home-deployable robotic systems. This emphasis aligns with broader trends toward improving health care accessibility, reducing supervision burden, and advancing patient-centered care.

Our findings are directionally consistent with evidence that robot-assisted upper-limb rehabilitation can deliver high-intensity, repetitive, task-specific practice associated with functional gains in stroke populations while reducing therapist physical burden when appropriately dosed [8-11]. Mixed reality guidance using HoloLens has demonstrated feasibility for spatial cues, hands-free visualization, and improved engagement in rehabilitation contexts [8-11]; this study extended this by coupling mixed reality tasking directly with instrumented, multimode robotic assistance and live telemetry for on-the-fly tuning. Previous telerehabilitation efforts and serious game platforms have reported enhanced motivation and remote monitoring [8-11]; in this study, the combination of mixed reality guidance and graded robotic support and resistance allowed us to preserve safety envelopes—defined as the predefined limits on kinematics and interaction forces that the system will not exceed (eg, workspace/range-of-motion boundaries and velocity)—while keeping sessions interactive. Finally, emerging DT frameworks emphasize remote visualization, logging, and data-driven personalization [8-11]; our cloud-synchronized twin mirrored joint states and interaction forces in real time, enabling therapist visibility and rapid parameter adjustments during the session. The unique value of the telerehabilitation platform lies in its innovative combination of DMRbotV3, mixed reality environments, and real-time data monitoring through DT technology. The system’s seamless integration allows for remote therapy sessions with therapist-guided adjustments via HoloLens 2 supported by low latency (approximately 90 ms) [45]. Gamified, task-oriented exercises in the mixed reality environment provide an engaging therapeutic experience while delivering quantitative feedback to both therapists and patients, enabling data-driven adjustments tailored to individual recovery needs. With dynamic, bidirectional communication facilitated by the Azure IoT Hub, the telerehabilitation platform offers a level of adaptability and responsiveness that distinguishes it from conventional rehabilitation systems.

The DMRbotV3 ensures user safety through multiple hardware and software features. Hardware safety features include adjustable mechanical stoppers for personalized joint limits; software safety features are added in the controller that include limiting the joints’ ranges of movements depending on the participant’s requirements and limiting the joints’ speed, torques, and voltage values, which are the final output of the controller and the command values to the motor. To take advantage of a secure cloud platform and support minimally supervised operation, the system was deployed on HIPAA (Health Insurance Portability and Accountability Act)-eligible Microsoft Azure services, using a data minimization strategy in which only deidentified device telemetry (forces, kinematics, and system events) was stored in Azure, and the reidentification key was maintained off-cloud on an encrypted, access-restricted drive [46]. Data were protected in transit via Transport Layer Security 1.2 and at rest via 256-bit Advanced Encryption Standard with HTTPS-only end points; access was governed by role-based access control on a least-privileged basis with multifactor authentication [47]. Device- and message-level security used distinct IoT identities and time-limited shared access signature tokens. Storage firewalls and private end points limited network exposure, and Azure Monitor provided continuous auditing and alerts. HoloLens 2 needs calibration, which is done automatically in seconds (<10 seconds) when a user wears it. When the internet connection was lost during a rehabilitation exercise, the connection between HoloLens 2 and DMRbotV3 was interrupted; the session stopped, and DMRbotV3 returned to its home position at a slow, controlled speed to ensure patient safety. After reconnecting to the internet, the connection between HoloLens 2 and the DMRbotV3 was re-established automatically, and the patient and therapist were notified. We tested the Azure cloud with a Microsoft server in the eastern United States (in Virginia, 1376 km from the BioRobotics laboratory in Milwaukee) to execute bidirectional data transfer between DMRbotV3 and HoloLens 2 in real time. The robotic rehabilitation system facilitates safe and controlled movement execution, providing real-time feedback on critical metrics such as patient arm force and robotic parameters. This dynamic feedback mechanism fosters a highly adaptable and responsive rehabilitation environment, effectively accommodating a wide range of recovery needs.

Unlike many commercial or laboratory-stage systems that emphasize single-mode assistance, 2D movements, or strictly in-clinic operation, our platform integrates 3 therapy modes (passive, active, and resistive) with mixed reality task guidance and a cloud-synchronized DT in a portable, desktop-mounted robot, explicitly designed for remotely supervised care. This end-to-end integration reduces the gap between engaging interfaces and precise physical assistance and addresses common gaps in prior systems (limited mode sets, constrained workspaces, and lack of telerehabilitation functionality) highlighted in the Introduction section [8-11]. Participants’ confidence in safety and their reports of autonomy and flexibility speak to the promise of minimally supervised or home-based use. Nonetheless, this study did not evaluate unsupervised patient or caregiver self-installation.

This study’s strengths include its innovative system design, which bridges critical gaps in traditional and emerging rehabilitation methods, and its focus on user-centered evaluation. This study assessed the system’s functionality and impact by collecting both qualitative feedback and quantitative data. However, the limited sample size and single-session design are notable weaknesses as they restrict the generalizability of the findings and preclude analysis of long-term therapeutic outcomes. Future studies should aim to conduct longitudinal evaluations with more extensive and diverse participant groups to validate the system’s effectiveness and usability over extended periods. Further research could explore the integration of artificial intelligence to enable adaptive learning based on patient progress, enhancing the system’s ability to deliver personalized therapy.

This study has several limitations that should be considered when interpreting the findings. First, the sample size was small and limited to a single site, which restricts the generalizability of the results to broader populations of survivors of stroke and rehabilitation practitioners. Second, the evaluation was conducted in a simulated laboratory environment rather than in patients’ homes or clinical settings, meaning that real-world usability, accessibility, and logistical challenges may not have been fully captured. Third, this study focused primarily on short-term usability during a single 2-hour session; longer-term use is necessary to evaluate sustained engagement, adherence, and potential therapeutic benefits. Fourth, while the customized usability questionnaire provided device-specific insights, the absence of standardized scales such as the System Usability Scale or Quebec User Evaluation of Satisfaction With Assistive Technology limits comparability with other studies in the field. Finally, this study emphasized subjective usability and safety outcomes but did not include objective clinical measures of motor recovery or functional improvement, which will be essential for future trials assessing therapeutic efficacy.

### Conclusions

This study introduced an innovative telerehabilitation platform integrated with a robotic rehabilitation system to provide a comprehensive framework for remote upper-limb therapy. The proposed system leverages advanced technologies, including mixed reality through HoloLens 2, gamification for engagement, and DT technology for real-time data monitoring and therapist interaction. A key strength of this system is its ability to deliver diverse therapy modes, such as passive, active, and resistive, allowing for tailored rehabilitation routines that adapt to individual patient needs and recovery stages. The experimental results demonstrated that the telerehabilitation platform offers a promising alternative to traditional in-person rehabilitation, addressing common barriers such as geographical and logistical constraints. Participant feedback reflected high satisfaction with the device’s usability, adaptability, and safety, suggesting the telerehabilitation platform’s potential to enhance engagement and adherence in home-based rehabilitation. Further research with a larger and more diverse participant sample, including control groups and extended intervention periods, would provide valuable insights into the system’s long-term effectiveness and comparative performance against traditional rehabilitation methods while identifying factors to enhance neurorehabilitation outcomes. In addition, integrating machine learning algorithms to analyze patient data and optimize therapy delivery presents an exciting opportunity to advance personalization and improve therapeutic efficacy, paving the way for more precise and impactful rehabilitation solutions.

## Abbreviations

DT: digital twin
HIPAA: Health Insurance Portability and Accountability Act
IoT: Internet of Things
MRTK3: Mixed Reality Toolkit 3
VR: virtual reality
